# (*E*)-4-Amino-*N*′-(2-hy­droxy-5-methyl­benzyl­idene)benzohydrazide

**DOI:** 10.1107/S1600536812027948

**Published:** 2012-06-23

**Authors:** Hadi Kargar, Reza Kia, Muhammad Nawaz Tahir

**Affiliations:** a Department of Chemistry, Payame Noor University, PO Box 19395-3697 Tehran, I. R. of IRAN; b Department of Chemistry, Science and Research Branch, Islamic Azad University, Tehran, Iran; c Department of Physics, University of Sargodha, Punjab, Pakistan

## Abstract

The asymmetric unit of the title compound, C_15_H_15_N_3_O_2_, comprises two crystallographically independent mol­ecules (*A* and *B*), each having an *E* conformation around the C=N bond. In each mol­ecule, there is an intra­molecular O—H⋯N hydrogen bond making an *S*(6) ring motif. The dihedral angles between the substituted phenyl rings are 17.49 (9) and 10.03 (9)°. In the crystal, N—H⋯O hydrogen bonds link neighbouring independent mol­ecules into infinite chains, of the type –*A*–*B*–*A*–*B*–, along the *a* axis, enclosing *R*_2_^1^(7) ring motifs. The chains are linked by N—H⋯O hydrogen bonds and C—H⋯O inter­actions, leading to the formation of a three-dimensional network.

## Related literature

For the coordination chemistry of Schiff base and hydrazone derivatives, see: Kucukguzel *et al.* (2006 ▶); Karthikeyan *et al.* (2006 ▶). For 4-amino­benzohydrazide-derived Schiff base structures, see: Xu (2012 ▶); Shi & Li (2012 ▶); Bakir & Green (2002 ▶); Kargar *et al.* (2012*a* ▶,*b* ▶). For standard bond lengths, see: Allen *et al.* (1987 ▶). For hydrogen-bond motifs, see: Bernstein *et al.* (1995 ▶).
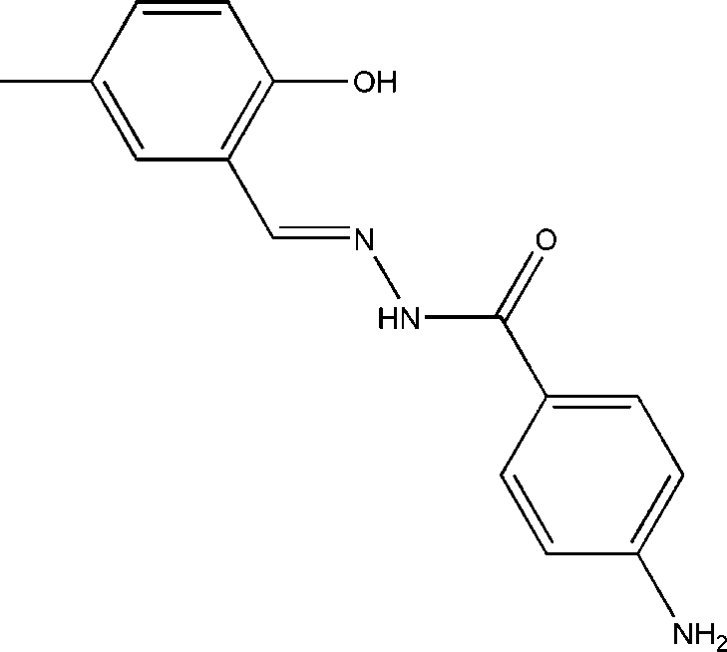


## Experimental

### 

#### Crystal data

C_15_H_15_N_3_O_2_
*M* *_r_* = 269.30
Triclinic, 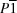

*a* = 10.2717 (8) Å
*b* = 11.5668 (10) Å
*c* = 11.9152 (9) Å
α = 94.544 (3)°
β = 100.583 (3)°
γ = 95.880 (3)°
*V* = 1377.28 (19) Å^3^
*Z* = 4
Mo *K*α radiation
μ = 0.09 mm^−1^
*T* = 291 K
0.30 × 0.25 × 0.24 mm

#### Data collection

Bruker SMART APEXII CCD area-detector diffractometer
Absorption correction: multi-scan (*SADABS*; Bruker, 2005 ▶) *T*_min_ = 0.974, *T*_max_ = 0.979
21355 measured reflections
6081 independent reflections
3917 reflections with *I* > 2σ(*I*)
*R*_int_ = 0.028

#### Refinement

*R*[*F* ^2^ > 2σ(*F* ^2^)] = 0.047
*wR*(*F* ^2^) = 0.137
*S* = 1.05
6081 reflections
365 parameters
H-atom parameters constrained
Δρ_max_ = 0.19 e Å^−3^
Δρ_min_ = −0.17 e Å^−3^

### 

Data collection: *APEX2* (Bruker, 2005 ▶); cell refinement: *SAINT* (Bruker, 2005 ▶); data reduction: *SAINT*; program(s) used to solve structure: *SHELXS97* (Sheldrick, 2008 ▶); program(s) used to refine structure: *SHELXL97* (Sheldrick, 2008 ▶); molecular graphics: *SHELXTL* (Sheldrick, 2008 ▶); software used to prepare material for publication: *SHELXTL* and *PLATON* (Spek, 2009 ▶).

## Supplementary Material

Crystal structure: contains datablock(s) global, I. DOI: 10.1107/S1600536812027948/su2458sup1.cif

Structure factors: contains datablock(s) I. DOI: 10.1107/S1600536812027948/su2458Isup2.hkl

Supplementary material file. DOI: 10.1107/S1600536812027948/su2458Isup3.cml

Additional supplementary materials:  crystallographic information; 3D view; checkCIF report

## Figures and Tables

**Table 1:** Hydrogen-bond geometry (Å, °)

*D*—H⋯*A*	*D*—H	H⋯*A*	*D*⋯*A*	*D*—H⋯*A*
O2—H2⋯N3	0.82	1.89	2.6079 (19)	145
O4—H4⋯N6	0.82	1.86	2.5826 (17)	146
N2—H1*N*2⋯O4^i^	0.94	2.01	2.9342 (18)	169
N5—H1*N*5⋯O1^ii^	0.95	1.93	2.8615 (17)	167
N1—H1*A*⋯O3^iii^	0.86	2.42	3.149 (2)	142
C12—H12*A*⋯O3^iv^	0.93	2.51	3.400 (2)	160
C17—H17*A*⋯O1^ii^	0.93	2.53	3.320 (2)	143

Symmetry codes: (i) 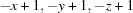; (ii) 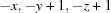; (iii) 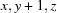; (iv) 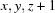.

**Fig. 1.**
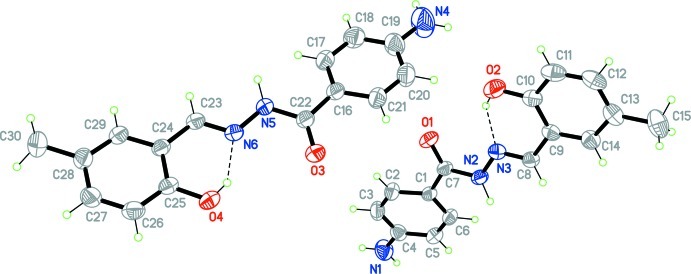
A view of the molecular structure of the two independent molecules of the title compound, showing 40% probability displacement ellipsoids and the atomic numbering. The intramolecular O-H···N hydrogen bonds are shown as dashed lines (see Table 1 for details).

**Fig. 2.**
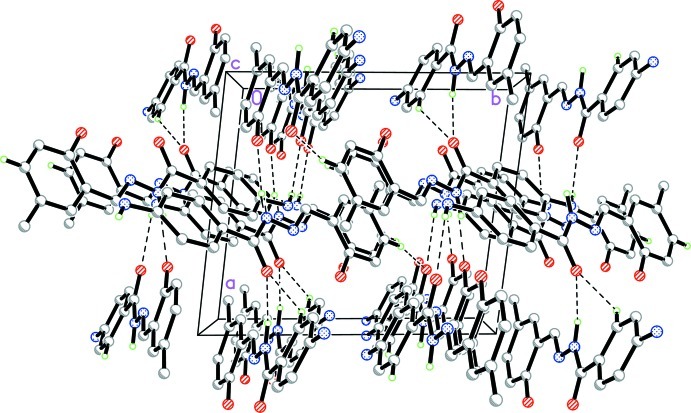
A view along the c axis of the crystal packing of the title compound. The N—H···O hydrogen bonds and the C—H···O interactions are shown as dashed lines (see Table 1 for details; Only the H atoms involved in these interactions are shown).

## supplementary crystallographic information

### Comment

Schiff bases are one of the most prevalent mixed-donor ligands in the field of coordination chemistry. They play an important role in the development of coordination chemistry related to catalysis and magnetism, and supramolecular architectures (Karthikeyan *et al.*, 2006; Kucukguzel *et al.*, 2006). Structures of Schiff bases derived from substituted 4-aminobenzohydrazide have been reported earlier (Xu, 2012; Shi & Li, 2012; Bakir & Green, 2002; Kargar *et al.* (2012**a*,b*). In order to explore the structure of new Schiff base derivatives, the title compound was prepared and characterized crystallographically.

The asymmetric unit of the title compound, Fig. 1, comprises two crystallographically independent molecules (A and B) both with an *E* conformation around the C═N bond. The bond lengths (Allen *et al.*, 1987) and angles are within normal ranges and are comparable to those reported for related structures (Xu, 2012; Shi & Li, 2012; Bakir & Green, 2002; Kargar *et al.* (2012**a*,b*). In each molecule there is an intramolecular O—H···N hydrogen bond making an *S(6)* ring motif. The dihedral angles between the substituted phenyl rings (C1-C6/C9-C14 in molecule A and C16-C21/C24-C29 in molecule B) are 17.49 (9) and 10.03 (9)°, respectively.

In the crystal, N—H···O hydrogen bonds (Table 1 and Fig. 2) link neighbouring independent molecules into infinite chains, of the type -A-B-A-B-, along the *a* axis, enclosing *R^1^_2_(7)* ring motifs (Bernstein *et al.*, 1995). The chains are linked by N-H···O hydrogen bonds and C—H···O interactions leading to the formation of a three-dimensional network (Table 1).

### Experimental

The title compound was synthesized by adding 1 mmol of methyl 4-aminobenzoate to a solution of 5-methylsalicylaldehyde (1 mmol) in methanol (30 ml). The mixture was refluxed with stirring for 30 min and after cooling to room temperature a light-yellow precipitate was filtered and washed with diethylether and dried in air. Yellow prismatic crystals of the title compound, suitable for *X*-ray structure analysis, were recrystallized from ethanol by slow evaporation of the solvents at room temperature over several days.

### Refinement

The N-bound H-atoms were located in a difference Fourier map and were constrained to ride on their parent N atoms with U_iso_(H) = 1.2U_eq_(N). The OH and C-bound H atoms were included in calculated positions and treated as riding atoms: O-H = 0.82 Å, C-H = 0.93 and 0.96 Å for CH and CH_3_ H atoms, respectively, with U_iso_(H) = k × U_eq_(O,C) where k = 1.5 for OH and CH_3_ H atoms and = 1.2 for other H atoms.

### Crystal data

**Table:**

C_15_H_15_N_3_O_2_	*Z* = 4
*M**_r_* = 269.30	*F*(000) = 568
Triclinic, *P*1	*D*_x_ = 1.299 Mg m^−^^3^
Hall symbol: -P 1	Mo *K*α radiation, λ = 0.71073 Å
*a* = 10.2717 (8) Å	Cell parameters from 1125 reflections
*b* = 11.5668 (10) Å	θ = 2.5–27.4°
*c* = 11.9152 (9) Å	µ = 0.09 mm^−^^1^
α = 94.544 (3)°	*T* = 291 K
β = 100.583 (3)°	Prism, yellow
γ = 95.880 (3)°	0.30 × 0.25 × 0.24 mm
*V* = 1377.28 (19) Å^3^	

### Data collection

**Table:**

Bruker SMART APEXII CCD area-detector diffractometer	6081 independent reflections
Radiation source: fine-focus sealed tube	3917 reflections with *I* > 2σ(*I*)
Graphite monochromator	*R*_int_ = 0.028
φ and ω scans	θ_max_ = 27.2°, θ_min_ = 1.8°
Absorption correction: multi-scan (*SADABS*; Bruker, 2005)	*h* = −13→13
*T*_min_ = 0.974, *T*_max_ = 0.979	*k* = −14→14
21355 measured reflections	*l* = −15→13

### Refinement

**Table:**

Refinement on *F*^2^	Primary atom site location: structure-invariant direct methods
Least-squares matrix: full	Secondary atom site location: difference Fourier map
*R*[*F*^2^ > 2σ(*F*^2^)] = 0.047	Hydrogen site location: inferred from neighbouring sites
*wR*(*F*^2^) = 0.137	H-atom parameters constrained
*S* = 1.05	*w* = 1/[σ^2^(*F*_o_^2^) + (0.0572*P*)^2^ + 0.2031*P*] where *P* = (*F*_o_^2^ + 2*F*_c_^2^)/3
6081 reflections	(Δ/σ)_max_ < 0.001
365 parameters	Δρ_max_ = 0.19 e Å^−^^3^
0 restraints	Δρ_min_ = −0.17 e Å^−^^3^

### Special details

**Table:**

Geometry. All esds (except the esd in the dihedral angle between two l.s. planes) are estimated using the full covariance matrix. The cell esds are taken into account individually in the estimation of esds in distances, angles and torsion angles; correlations between esds in cell parameters are only used when they are defined by crystal symmetry. An approximate (isotropic) treatment of cell esds is used for estimating esds involving l.s. planes.
Refinement. Refinement of F^2^ against ALL reflections. The weighted R-factor wR and goodness of fit S are based on F^2^, conventional R-factors R are based on F, with F set to zero for negative F^2^. The threshold expression of F^2^ > 2sigma(F^2^) is used only for calculating R-factors(gt) etc. and is not relevant to the choice of reflections for refinement. R-factors based on F^2^ are statistically about twice as large as those based on F, and R- factors based on ALL data will be even larger.

### Fractional atomic coordinates and isotropic or equivalent isotropic displacement parameters (Å^2^)

**Table:**

	*x*	*y*	*z*	*U*_iso_*/*U*_eq_	
C1	0.41397 (14)	0.93883 (14)	0.73216 (13)	0.0416 (4)	
C2	0.34820 (17)	0.95319 (16)	0.62200 (14)	0.0525 (4)	
H2A	0.2819	0.8953	0.5835	0.063*	
C3	0.37828 (18)	1.04928 (17)	0.56949 (15)	0.0580 (5)	
H3A	0.3332	1.0558	0.4955	0.070*	
C4	0.47549 (17)	1.13830 (16)	0.62444 (14)	0.0505 (4)	
C5	0.54375 (16)	1.12428 (15)	0.73354 (14)	0.0507 (4)	
H5A	0.6107	1.1819	0.7714	0.061*	
C6	0.51343 (15)	1.02622 (15)	0.78609 (14)	0.0467 (4)	
H6A	0.5604	1.0183	0.8591	0.056*	
C7	0.36676 (14)	0.83795 (14)	0.78761 (13)	0.0426 (4)	
C8	0.48321 (16)	0.68945 (14)	1.02368 (14)	0.0456 (4)	
H8A	0.5691	0.7281	1.0467	0.055*	
C9	0.43816 (16)	0.59517 (14)	1.08529 (14)	0.0446 (4)	
C10	0.31463 (17)	0.52710 (15)	1.04709 (15)	0.0515 (4)	
C11	0.2798 (2)	0.43601 (16)	1.10815 (19)	0.0650 (5)	
H11A	0.1992	0.3889	1.0822	0.078*	
C12	0.3624 (2)	0.41382 (16)	1.20672 (18)	0.0648 (5)	
H12A	0.3353	0.3532	1.2473	0.078*	
C13	0.4847 (2)	0.47943 (16)	1.24696 (15)	0.0584 (5)	
C14	0.51992 (18)	0.56874 (15)	1.18424 (15)	0.0525 (4)	
H14A	0.6023	0.6134	1.2093	0.063*	
C15	0.5763 (3)	0.4523 (2)	1.35265 (18)	0.0848 (7)	
H15A	0.6484	0.4149	1.3308	0.127*	
H15B	0.5276	0.4011	1.3942	0.127*	
H15C	0.6115	0.5234	1.4005	0.127*	
C16	0.06408 (16)	0.30308 (14)	0.48957 (14)	0.0469 (4)	
C17	−0.05809 (17)	0.34582 (17)	0.48283 (16)	0.0572 (5)	
H17A	−0.1126	0.3465	0.4114	0.069*	
C18	−0.1001 (2)	0.38722 (18)	0.57992 (17)	0.0681 (5)	
H18A	−0.1829	0.4145	0.5733	0.082*	
C19	−0.0209 (2)	0.38872 (19)	0.68691 (17)	0.0691 (5)	
C20	0.1007 (2)	0.3453 (2)	0.69390 (17)	0.0709 (6)	
H20A	0.1551	0.3446	0.7654	0.085*	
C21	0.14190 (18)	0.30375 (17)	0.59809 (15)	0.0590 (5)	
H21A	0.2241	0.2751	0.6053	0.071*	
C22	0.11833 (16)	0.26239 (15)	0.38986 (14)	0.0466 (4)	
C23	−0.00670 (15)	0.18859 (14)	0.09605 (14)	0.0458 (4)	
H23A	−0.0949	0.2024	0.0929	0.055*	
C24	0.03676 (15)	0.14797 (14)	−0.00741 (13)	0.0433 (4)	
C25	0.16631 (15)	0.12065 (14)	−0.00588 (14)	0.0454 (4)	
C26	0.20213 (17)	0.07912 (16)	−0.10588 (16)	0.0576 (5)	
H26A	0.2885	0.0610	−0.1046	0.069*	
C27	0.11136 (18)	0.06414 (16)	−0.20780 (16)	0.0597 (5)	
H27A	0.1376	0.0364	−0.2746	0.072*	
C28	−0.01883 (18)	0.08963 (16)	−0.21308 (15)	0.0557 (5)	
C29	−0.05284 (16)	0.13124 (15)	−0.11215 (14)	0.0495 (4)	
H29A	−0.1394	0.1490	−0.1139	0.059*	
C30	−0.1168 (2)	0.0702 (2)	−0.32422 (16)	0.0785 (6)	
H30A	−0.1578	0.1401	−0.3377	0.118*	
H30B	−0.1841	0.0073	−0.3208	0.118*	
H30C	−0.0715	0.0509	−0.3856	0.118*	
N1	0.50273 (18)	1.23589 (15)	0.57157 (14)	0.0716 (5)	
H1A	0.4596	1.2425	0.5038	0.086*	
H1B	0.5630	1.2907	0.6060	0.086*	
N2	0.45092 (13)	0.80883 (12)	0.88017 (11)	0.0473 (3)	
H1N2	0.5423	0.8349	0.8962	0.057*	
N3	0.40512 (13)	0.71958 (12)	0.93778 (12)	0.0474 (3)	
N4	−0.0612 (2)	0.4296 (2)	0.78470 (17)	0.1166 (8)	
H4A	−0.1376	0.4551	0.7801	0.140*	
H4B	−0.0101	0.4297	0.8506	0.140*	
N5	0.03008 (13)	0.24063 (13)	0.28828 (11)	0.0509 (4)	
H1N5	−0.0634	0.2399	0.2848	0.061*	
N6	0.07587 (12)	0.20552 (12)	0.19168 (11)	0.0464 (3)	
O1	0.25527 (10)	0.78344 (11)	0.75600 (10)	0.0585 (3)	
O2	0.22764 (12)	0.54605 (12)	0.95216 (12)	0.0704 (4)	
H2	0.2591	0.6017	0.9231	0.106*	
O3	0.23698 (11)	0.24990 (12)	0.39557 (10)	0.0596 (3)	
O4	0.25969 (10)	0.13154 (11)	0.09292 (10)	0.0552 (3)	
H4	0.2266	0.1546	0.1469	0.083*	

### Atomic displacement parameters (Å^2^)

**Table:**

	*U*^11^	*U*^22^	*U*^33^	*U*^12^	*U*^13^	*U*^23^
C1	0.0320 (8)	0.0510 (10)	0.0423 (9)	0.0098 (7)	0.0070 (7)	0.0025 (7)
C2	0.0493 (10)	0.0606 (11)	0.0427 (10)	0.0039 (8)	−0.0005 (8)	0.0006 (8)
C3	0.0627 (11)	0.0695 (13)	0.0394 (10)	0.0108 (9)	0.0012 (8)	0.0074 (9)
C4	0.0528 (10)	0.0540 (11)	0.0490 (10)	0.0141 (8)	0.0146 (8)	0.0099 (8)
C5	0.0425 (9)	0.0509 (10)	0.0554 (11)	0.0041 (7)	0.0035 (8)	0.0024 (8)
C6	0.0385 (8)	0.0546 (10)	0.0448 (9)	0.0091 (7)	0.0001 (7)	0.0065 (8)
C7	0.0316 (8)	0.0521 (10)	0.0436 (9)	0.0071 (7)	0.0065 (7)	−0.0001 (7)
C8	0.0412 (9)	0.0468 (9)	0.0474 (9)	−0.0015 (7)	0.0095 (7)	0.0034 (7)
C9	0.0476 (9)	0.0415 (9)	0.0466 (9)	0.0020 (7)	0.0176 (8)	0.0011 (7)
C10	0.0489 (10)	0.0495 (10)	0.0574 (11)	0.0009 (8)	0.0183 (9)	0.0002 (8)
C11	0.0615 (12)	0.0503 (11)	0.0861 (15)	−0.0065 (9)	0.0314 (11)	0.0020 (10)
C12	0.0815 (14)	0.0470 (11)	0.0766 (14)	0.0047 (10)	0.0428 (12)	0.0118 (10)
C13	0.0804 (13)	0.0466 (10)	0.0548 (11)	0.0138 (9)	0.0259 (10)	0.0076 (8)
C14	0.0581 (10)	0.0463 (10)	0.0529 (10)	0.0014 (8)	0.0132 (8)	0.0041 (8)
C15	0.122 (2)	0.0713 (14)	0.0660 (14)	0.0185 (13)	0.0191 (13)	0.0254 (11)
C16	0.0413 (9)	0.0496 (10)	0.0476 (10)	−0.0005 (7)	0.0056 (7)	0.0073 (8)
C17	0.0481 (10)	0.0683 (12)	0.0524 (11)	0.0077 (9)	0.0026 (8)	0.0037 (9)
C18	0.0568 (11)	0.0783 (14)	0.0694 (13)	0.0172 (10)	0.0118 (10)	−0.0016 (11)
C19	0.0748 (14)	0.0764 (14)	0.0577 (12)	0.0163 (11)	0.0172 (11)	−0.0033 (10)
C20	0.0685 (13)	0.0935 (16)	0.0475 (11)	0.0181 (11)	0.0021 (10)	−0.0009 (10)
C21	0.0513 (10)	0.0749 (13)	0.0485 (11)	0.0110 (9)	0.0023 (8)	0.0044 (9)
C22	0.0393 (9)	0.0522 (10)	0.0464 (10)	−0.0011 (7)	0.0042 (7)	0.0109 (8)
C23	0.0337 (8)	0.0553 (10)	0.0484 (10)	0.0044 (7)	0.0071 (7)	0.0073 (8)
C24	0.0355 (8)	0.0450 (9)	0.0474 (9)	−0.0010 (7)	0.0053 (7)	0.0064 (7)
C25	0.0357 (8)	0.0454 (9)	0.0523 (10)	−0.0022 (7)	0.0055 (7)	0.0050 (7)
C26	0.0436 (9)	0.0596 (11)	0.0700 (12)	0.0034 (8)	0.0177 (9)	−0.0029 (9)
C27	0.0588 (11)	0.0628 (12)	0.0567 (11)	−0.0015 (9)	0.0187 (9)	−0.0044 (9)
C28	0.0565 (11)	0.0569 (11)	0.0494 (10)	−0.0032 (8)	0.0057 (8)	0.0024 (8)
C29	0.0377 (8)	0.0581 (11)	0.0504 (10)	0.0030 (7)	0.0037 (7)	0.0061 (8)
C30	0.0803 (14)	0.0929 (17)	0.0531 (12)	−0.0014 (12)	0.0007 (11)	−0.0035 (11)
N1	0.0848 (12)	0.0661 (11)	0.0637 (10)	0.0052 (9)	0.0104 (9)	0.0193 (8)
N2	0.0338 (7)	0.0550 (9)	0.0521 (8)	−0.0008 (6)	0.0053 (6)	0.0145 (7)
N3	0.0425 (7)	0.0497 (8)	0.0504 (8)	−0.0001 (6)	0.0121 (7)	0.0066 (7)
N4	0.1149 (17)	0.170 (2)	0.0722 (13)	0.0597 (17)	0.0252 (12)	−0.0143 (14)
N5	0.0348 (7)	0.0746 (10)	0.0412 (8)	0.0023 (6)	0.0060 (6)	0.0026 (7)
N6	0.0358 (7)	0.0556 (9)	0.0475 (8)	0.0006 (6)	0.0083 (6)	0.0077 (6)
O1	0.0343 (6)	0.0699 (8)	0.0650 (8)	−0.0040 (5)	−0.0003 (5)	0.0048 (6)
O2	0.0516 (7)	0.0749 (10)	0.0781 (9)	−0.0115 (6)	0.0052 (7)	0.0092 (7)
O3	0.0382 (7)	0.0875 (9)	0.0533 (7)	0.0084 (6)	0.0064 (5)	0.0136 (6)
O4	0.0332 (6)	0.0699 (8)	0.0591 (8)	0.0054 (5)	0.0015 (5)	0.0041 (6)

### Geometric parameters (Å, º)

**Table:**

C1—C6	1.387 (2)	C18—C19	1.378 (3)
C1—C2	1.392 (2)	C18—H18A	0.9300
C1—C7	1.469 (2)	C19—N4	1.372 (3)
C2—C3	1.354 (2)	C19—C20	1.384 (3)
C2—H2A	0.9300	C20—C21	1.359 (3)
C3—C4	1.389 (2)	C20—H20A	0.9300
C3—H3A	0.9300	C21—H21A	0.9300
C4—N1	1.365 (2)	C22—O3	1.2320 (19)
C4—C5	1.389 (2)	C22—N5	1.361 (2)
C5—C6	1.374 (2)	C23—N6	1.2773 (19)
C5—H5A	0.9300	C23—C24	1.447 (2)
C6—H6A	0.9300	C23—H23A	0.9300
C7—O1	1.2274 (18)	C24—C29	1.395 (2)
C7—N2	1.3585 (19)	C24—C25	1.396 (2)
C8—N3	1.2745 (19)	C25—O4	1.3633 (18)
C8—C9	1.446 (2)	C25—C26	1.376 (2)
C8—H8A	0.9300	C26—C27	1.375 (2)
C9—C14	1.391 (2)	C26—H26A	0.9300
C9—C10	1.401 (2)	C27—C28	1.390 (3)
C10—O2	1.354 (2)	C27—H27A	0.9300
C10—C11	1.381 (3)	C28—C29	1.377 (2)
C11—C12	1.375 (3)	C28—C30	1.495 (2)
C11—H11A	0.9300	C29—H29A	0.9300
C12—C13	1.382 (3)	C30—H30A	0.9600
C12—H12A	0.9300	C30—H30B	0.9600
C13—C14	1.379 (2)	C30—H30C	0.9600
C13—C15	1.503 (3)	N1—H1A	0.8600
C14—H14A	0.9300	N1—H1B	0.8600
C15—H15A	0.9600	N2—N3	1.3746 (18)
C15—H15B	0.9600	N2—H1N2	0.9357
C15—H15C	0.9600	N4—H4A	0.8600
C16—C17	1.386 (2)	N4—H4B	0.8600
C16—C21	1.389 (2)	N5—N6	1.3693 (18)
C16—C22	1.467 (2)	N5—H1N5	0.9526
C17—C18	1.375 (3)	O2—H2	0.8200
C17—H17A	0.9300	O4—H4	0.8200
			
C6—C1—C2	117.47 (15)	C19—C18—H18A	119.6
C6—C1—C7	123.98 (14)	N4—C19—C18	121.6 (2)
C2—C1—C7	118.34 (14)	N4—C19—C20	120.3 (2)
C3—C2—C1	121.66 (16)	C18—C19—C20	118.12 (18)
C3—C2—H2A	119.2	C21—C20—C19	121.11 (18)
C1—C2—H2A	119.2	C21—C20—H20A	119.4
C2—C3—C4	120.99 (16)	C19—C20—H20A	119.4
C2—C3—H3A	119.5	C20—C21—C16	121.48 (18)
C4—C3—H3A	119.5	C20—C21—H21A	119.3
N1—C4—C3	120.40 (16)	C16—C21—H21A	119.3
N1—C4—C5	121.57 (17)	O3—C22—N5	120.98 (16)
C3—C4—C5	118.03 (16)	O3—C22—C16	122.88 (15)
C6—C5—C4	120.71 (16)	N5—C22—C16	116.13 (14)
C6—C5—H5A	119.6	N6—C23—C24	120.07 (14)
C4—C5—H5A	119.6	N6—C23—H23A	120.0
C5—C6—C1	121.12 (15)	C24—C23—H23A	120.0
C5—C6—H6A	119.4	C29—C24—C25	117.95 (15)
C1—C6—H6A	119.4	C29—C24—C23	120.09 (14)
O1—C7—N2	120.24 (15)	C25—C24—C23	121.93 (14)
O1—C7—C1	122.84 (14)	O4—C25—C26	118.01 (14)
N2—C7—C1	116.88 (13)	O4—C25—C24	122.05 (15)
N3—C8—C9	119.98 (15)	C26—C25—C24	119.92 (15)
N3—C8—H8A	120.0	C27—C26—C25	120.60 (16)
C9—C8—H8A	120.0	C27—C26—H26A	119.7
C14—C9—C10	118.37 (15)	C25—C26—H26A	119.7
C14—C9—C8	119.70 (15)	C26—C27—C28	121.37 (17)
C10—C9—C8	121.92 (15)	C26—C27—H27A	119.3
O2—C10—C11	118.23 (16)	C28—C27—H27A	119.3
O2—C10—C9	122.92 (16)	C29—C28—C27	117.21 (16)
C11—C10—C9	118.84 (17)	C29—C28—C30	122.23 (18)
C12—C11—C10	121.07 (18)	C27—C28—C30	120.55 (18)
C12—C11—H11A	119.5	C28—C29—C24	122.95 (16)
C10—C11—H11A	119.5	C28—C29—H29A	118.5
C11—C12—C13	121.56 (18)	C24—C29—H29A	118.5
C11—C12—H12A	119.2	C28—C30—H30A	109.5
C13—C12—H12A	119.2	C28—C30—H30B	109.5
C14—C13—C12	116.99 (18)	H30A—C30—H30B	109.5
C14—C13—C15	121.74 (19)	C28—C30—H30C	109.5
C12—C13—C15	121.25 (18)	H30A—C30—H30C	109.5
C13—C14—C9	123.14 (17)	H30B—C30—H30C	109.5
C13—C14—H14A	118.4	C4—N1—H1A	120.0
C9—C14—H14A	118.4	C4—N1—H1B	120.0
C13—C15—H15A	109.5	H1A—N1—H1B	120.0
C13—C15—H15B	109.5	C7—N2—N3	117.71 (13)
H15A—C15—H15B	109.5	C7—N2—H1N2	122.5
C13—C15—H15C	109.5	N3—N2—H1N2	118.4
H15A—C15—H15C	109.5	C8—N3—N2	118.76 (13)
H15B—C15—H15C	109.5	C19—N4—H4A	120.0
C17—C16—C21	117.31 (16)	C19—N4—H4B	120.0
C17—C16—C22	124.35 (15)	H4A—N4—H4B	120.0
C21—C16—C22	118.28 (15)	C22—N5—N6	118.69 (13)
C18—C17—C16	121.16 (17)	C22—N5—H1N5	121.4
C18—C17—H17A	119.4	N6—N5—H1N5	119.5
C16—C17—H17A	119.4	C23—N6—N5	118.36 (13)
C17—C18—C19	120.81 (18)	C10—O2—H2	109.5
C17—C18—H18A	119.6	C25—O4—H4	109.5
			
C6—C1—C2—C3	−0.9 (2)	C17—C18—C19—C20	−1.3 (3)
C7—C1—C2—C3	174.06 (16)	N4—C19—C20—C21	179.6 (2)
C1—C2—C3—C4	−0.7 (3)	C18—C19—C20—C21	0.9 (3)
C2—C3—C4—N1	−178.50 (17)	C19—C20—C21—C16	0.0 (3)
C2—C3—C4—C5	1.9 (3)	C17—C16—C21—C20	−0.4 (3)
N1—C4—C5—C6	178.96 (16)	C22—C16—C21—C20	176.92 (18)
C3—C4—C5—C6	−1.4 (3)	C17—C16—C22—O3	163.12 (17)
C4—C5—C6—C1	−0.2 (3)	C21—C16—C22—O3	−14.1 (3)
C2—C1—C6—C5	1.3 (2)	C17—C16—C22—N5	−15.7 (2)
C7—C1—C6—C5	−173.31 (15)	C21—C16—C22—N5	167.10 (15)
C6—C1—C7—O1	155.69 (16)	N6—C23—C24—C29	−178.99 (15)
C2—C1—C7—O1	−18.9 (2)	N6—C23—C24—C25	3.2 (2)
C6—C1—C7—N2	−21.9 (2)	C29—C24—C25—O4	−178.41 (14)
C2—C1—C7—N2	163.53 (14)	C23—C24—C25—O4	−0.5 (2)
N3—C8—C9—C14	−174.96 (15)	C29—C24—C25—C26	0.3 (2)
N3—C8—C9—C10	6.4 (2)	C23—C24—C25—C26	178.13 (16)
C14—C9—C10—O2	179.69 (16)	O4—C25—C26—C27	178.73 (15)
C8—C9—C10—O2	−1.6 (3)	C24—C25—C26—C27	0.0 (3)
C14—C9—C10—C11	−0.8 (2)	C25—C26—C27—C28	−0.4 (3)
C8—C9—C10—C11	177.92 (16)	C26—C27—C28—C29	0.4 (3)
O2—C10—C11—C12	−178.53 (17)	C26—C27—C28—C30	−178.63 (17)
C9—C10—C11—C12	1.9 (3)	C27—C28—C29—C24	−0.1 (3)
C10—C11—C12—C13	−1.8 (3)	C30—C28—C29—C24	178.90 (17)
C11—C12—C13—C14	0.4 (3)	C25—C24—C29—C28	−0.2 (2)
C11—C12—C13—C15	−178.03 (18)	C23—C24—C29—C28	−178.10 (16)
C12—C13—C14—C9	0.7 (3)	O1—C7—N2—N3	−1.8 (2)
C15—C13—C14—C9	179.17 (17)	C1—C7—N2—N3	175.79 (13)
C10—C9—C14—C13	−0.5 (3)	C9—C8—N3—N2	−179.45 (14)
C8—C9—C14—C13	−179.26 (16)	C7—N2—N3—C8	178.80 (14)
C21—C16—C17—C18	0.0 (3)	O3—C22—N5—N6	−0.6 (2)
C22—C16—C17—C18	−177.14 (17)	C16—C22—N5—N6	178.23 (14)
C16—C17—C18—C19	0.8 (3)	C24—C23—N6—N5	−178.08 (14)
C17—C18—C19—N4	−179.9 (2)	C22—N5—N6—C23	−177.96 (14)

### Hydrogen-bond geometry (Å, º)

**Table:**

*D*—H···*A*	*D*—H	H···*A*	*D*···*A*	*D*—H···*A*
O2—H2···N3	0.82	1.89	2.6079 (19)	145
O4—H4···N6	0.82	1.86	2.5826 (17)	146
N2—H1*N*2···O4^i^	0.94	2.01	2.9342 (18)	169
N5—H1*N*5···O1^ii^	0.95	1.93	2.8615 (17)	167
N1—H1*A*···O3^iii^	0.86	2.42	3.149 (2)	142
C12—H12*A*···O3^iv^	0.93	2.51	3.400 (2)	160
C17—H17*A*···O1^ii^	0.93	2.53	3.320 (2)	143

Symmetry codes: (i) −*x*+1, −*y*+1, −*z*+1; (ii) −*x*, −*y*+1, −*z*+1; (iii) *x*, *y*+1, *z*; (iv) *x*, *y*, *z*+1.
